# Operationalizing Co-Design in Exercise Interventions with Indigenous Peoples in Australia: Development and Cultural Adaptation of the PrIDE Tools

**DOI:** 10.3390/ijerph23020252

**Published:** 2026-02-17

**Authors:** Morwenna Kirwan, Connie Henson, Blade Bancroft-Duroux, Kerri Colegate, Cheryl Taylor, David Meharg, Neale Cohen, Kylie Gwynne

**Affiliations:** 1 Nura Gili: Centre for Indigenous Programs, University of New South Wales (UNSW), Kensington, NSW 2052, Australia; c.henson@unsw.edu.au (C.H.); b.bancroft_duroux@unsw.edu.au (B.B.-D.); k.colegate@unsw.edu.au (K.C.); kylie.gwynne@unsw.edu.au (K.G.); 2 Faculty of Medicine, Health and Human Sciences, Macquarie University, North Ryde, NSW 2109, Australia; 3 Djilba Disability Services, 76 Champion Drive, Seville Grove, WA 6112, Australia; 4 School of Population Health, Faculty of Medicine and Health, University of New South Wales (UNSW), Kensington, NSW 2052, Australia; d.meharg@unsw.edu.au; 5 Blakcademy, Faculty of Medicine and Health, University of New South Wales (UNSW), Kensington, NSW 2052, Australia; 6 Baker Heart and Diabetes Institute, 75 Commercial Road, Melbourne, VIC 3004, Australia; neale.cohen@baker.edu.au

**Keywords:** Indigenous Australians, co-design, cultural adaptation, group exercise, type 2 diabetes, cardiovascular disease, clinical yarning, behavior change, implementation science, quality appraisal

## Abstract

**Highlights:**

**Public health relevance—How does this work relate to a public health issue?**

- Indigenous Australians experience a disproportionate burden of type 2 diabetes and cardiovascular disease, with earlier onset, higher prevalence, and greater complications than other Australians, reflecting the profound impacts of colonization and ongoing structural inequities.
- Limited peer-reviewed evidence exists on culturally adapted exercise interventions co-designed with Indigenous Australians living with type 2 diabetes; substantial gaps remain in transparent reporting of Indigenous governance, cultural adaptation processes, and behavioral mechanisms.

**Public health significance—Why is this work of significance to public health?**

- This study advances the transparent documentation of systematic cultural adaptation through Indigenous-governed co-design, applying structured frameworks (CHRI Model, MADI, CREATE Quality Appraisal Tool, COM-B model) to address critical gaps in reproducibility and knowledge translation.
- Indigenous-governed co-design processes centered Indigenous knowledges and leadership beyond what consultation-based approaches achieve, demonstrating how behavior change mechanisms can be operationalized in culturally congruent ways that honor Indigenous epistemologies while maintaining evidence-based effectiveness.

**Public health implications—What are the key implications or messages for practitioners, policy makers and/or researchers in public health?**

- Health services seeking to develop culturally safe exercise interventions can use the PrIDE co-design methodology as a model, with transparent MADI documentation providing detailed guidance for systematic cultural adaptation and the Success Plan demonstrating the integration of clinical yarning into routine practice.
- Researchers and policymakers should prioritize Indigenous leadership and governance in systematic cultural adaptation using established frameworks, recognizing that documenting development processes is as critical as evaluating outcomes for achieving cultural safety, community trust, and scalability of health interventions.

**Abstract:**

Indigenous Australians experience a disproportionate burden of type 2 diabetes mellitus and cardiovascular disease. While clinician-led, community-based exercise programs are effective in general populations, limited peer-reviewed evidence is available describing culturally adapted exercise interventions with Indigenous Australians that transparently reports governance, cultural adaptation, and theoretical design. This paper reports the co-design and development of tools for the Preventing Indigenous Cardiovascular Disease and Diabetes through Exercise (PrIDE) study, an adaptation of the Beat It program that incorporates wearable technology. Using the Co-design Health Research and Innovation Model, four tools were developed with Indigenous governance through a Consumer Advisory Group and a project-specific Consumer User Panel. Three tools were culturally adapted—the PrIDE Exercise Program, the Strong Spirit Strong Self self-efficacy assessment, and Keep Your Heart Strong educational materials—and a newly developed tool, the Success Plan. Cultural adaptations were prospectively documented using the Model for Adaptation Design and Impact, and all tools were assessed using the Aboriginal and Torres Strait Islander Quality Appraisal Tool. Behavior change mechanisms were mapped using the COM-B model. This paper provides transparent documentation of culturally adapted theory-informed tool development to support reproducibility and knowledge translation. The evaluation of effectiveness, acceptability, and psychometric properties will be reported following PrIDE implementation.

## 1. Introduction

Aboriginal and Torres Strait Islander peoples (hereafter referred to as Indigenous Australians) belong to the world’s oldest continuing cultures, with deep knowledge systems and strong community connections that have sustained health and wellbeing for millennia. However, the impacts of colonization and ongoing structural inequities have resulted in a disproportionate burden of type 2 diabetes (T2DM) and cardiovascular disease (CVD), characterized by earlier onset, higher prevalence, and greater complications than other Australians [1,2,3,4]. These disparities reflect the profound impact of social, cultural, historical, and structural determinants of health [5]. Addressing these health inequities through culturally safe, community-led approaches is essential for advancing health equity and honoring Aboriginal and Torres Strait Islander peoples’ right to health and self-determination [6,7,8].

Regular, structured exercise is central to T2DM management and CVD prevention [9,10]. Clinician-led, community-based group exercise interventions have improved glycemic control, physical function, cardiovascular risk factors, and psychosocial outcomes in adults with T2DM, indicating effectiveness in general populations [10,11]. However, this evidence cannot be assumed to transfer directly to Indigenous populations [12,13,14]. A systematic review of community-based exercise interventions with Indigenous peoples from high-income colonized countries managing T2DM identified limited published evidence, with substantial heterogeneity in design, delivery, and reporting [15].

International evidence is limited but suggests promise. In one supervised group exercise intervention with Indigenous Polynesian adults with T2DM, participants demonstrated improvements in cardiometabolic outcomes and quality of life [16,17]. However, this study emphasized outcomes rather than documenting development processes, Indigenous governance, or cultural adaptation approaches, limiting reproducibility and knowledge translation to other Indigenous populations. This scarcity is reinforced by a systematic review of exercise-based interventions for Indigenous adults, which – although focused on chronic lung disease rather than T2DM—identified only one eligible study and highlighted inconsistent reporting of Indigenous governance, engagement, and cultural safety processes [18]. These findings suggest that limited transparency in co-design and governance is a broader issue in Indigenous exercise intervention research, extending beyond specific disease contexts.

Several exercise programs involving Indigenous Australians are described in the gray literature; however, few report peer-reviewed outcomes or provide sufficiently detailed, repeatable methods to support replication [19,20]. One notable exception is the Ironbark Program [21,22,23], a community-based fall prevention intervention for older Indigenous Australians incorporating group exercise and yarning. The program demonstrated improvements in physical function and high acceptability. While Ironbark provides an important example of culturally safe exercise delivery, its primary focus was feasibility and outcomes rather than systematic documentation of Indigenous-governed cultural adaptation processes or behavior change mechanisms. Together, this literature highlights a critical gap in exercise interventions developed with Indigenous Australians living with T2DM, where transparent, replicable documentation of cultural adaptation processes—particularly when grounded in behavioral theory—remains largely absent.

Against this background, the national Beat It program provided a strong evidence base for cultural adaptation. The Diabetes Australia program represents one of Australia’s most established evidence-based exercise interventions for adults with T2DM. Delivered as an eight-week supervised program by Accredited Exercise Physiologists (AEPs), the Beat It program has been found to improve physical function, strength, and psychosocial outcomes [24], with benefits maintained at 12-month follow-up [25]. The program has proven adaptable across diverse contexts, including online delivery [26] and cultural adaptation for Mandarin-speaking Chinese Australians [27]. Qualitative research identified core mechanisms supporting program success: clinician supervision, group cohesion, capability building, individualized adaptation, and delivery flexibility [28]. This body of work establishes Beat It as a mature, evidence-based program with well-articulated mechanisms.

Co-design has emerged as an effective method for developing culturally safe, acceptable, and effective health interventions [29]. Recent T2DM initiatives demonstrate the value of privileging Indigenous knowledge systems, lived experience, and governance throughout program development [30,31]. Internationally the OL@-OR@ mHealth program co-designed with Māori and Pasifika communities demonstrated that transparent Indigenous-governed co-design enhanced acceptability, engagement, and trust, even when short-term clinical effects were modest [32]. This finding highlights an important principle: documenting development processes matters as much as evaluating outcomes, because cultural safety and community trust are prerequisites for effectiveness and scalability [6,7]. A systematic review of Indigenous health interventions found frequent claims of cultural adaptation; however, Indigenous leadership, governance, and deep participation in intervention design were uncommon, with many studies relying on consultation rather than shared decision-making [14].

Despite these advances, substantial gaps remain. Many interventions labeled “co-designed” provide limited methodological detail on cultural adaptation decisions, how Indigenous governance shaped content, or how Indigenous knowledge systems informed theoretical mechanisms [29], a limitation also noted in a recent systematic review of Indigenous health intervention research [14]. The Preventing Indigenous CVD and Diabetes through Exercise (PrIDE) study was developed to address these gaps. The PrIDE study represents a co-designed adaptation of Beat It incorporating wearable technology for T2DM prevention and management and CVD risk reduction [33].

This paper reports the co-design and development of four PrIDE tools: the culturally adapted PrIDE Exercise Program, the Strong Spirit Strong Self self-efficacy assessment, Keep Your Heart Strong educational materials, and the Success Plan. In the PrIDE study protocol, these tools were identified as Phase 1 outputs (1, 3, and 5) [33]. By providing transparent documentation of cultural adaptation processes and theory-informed design decisions, this work offers practical guidance for operationalizing co-design of evidence-based exercise interventions with Indigenous Australians.

## 2. Materials and Methods

### 2.1. Study Design and Governance

This study employed a co-design approach underpinned by the Co-design Health Research and Innovation (CHRI) model [34], which emphasizes collective impact, power sharing, and resource sharing throughout the research process. The CHRI model privileges Indigenous perspectives and knowledges, recognizes community contributions meaningfully, and considers sustainability from the outset [34].

Indigenous governance was established at two levels. First, the CHRI Consumer Advisory Group (CAG), chaired by Associate Professor Cara Cross, a Worimi/Biripai (North Coast of New South Wales) Indigenous Australian woman, provided overarching governance across all CHRI research projects, meeting quarterly to review research plans and ensure consumer-focused, accessible, and meaningful research. Second, a project-specific Consumer User Panel (CUP) was established to provide dedicated cultural oversight and community voice specifically for the PrIDE study. The CUP comprised four Aboriginal and/or Torres Strait Islander members with deep community connections, representing diverse nations and bringing varied perspectives across age, gender, and health experiences. The CUP provided cultural guidance throughout tool development, including input into design, review of materials for clarity and cultural safety, and ensuring local cultural and community perspectives were centered.

### 2.2. The CHRI Model of Co-Design

**Figure 1:**
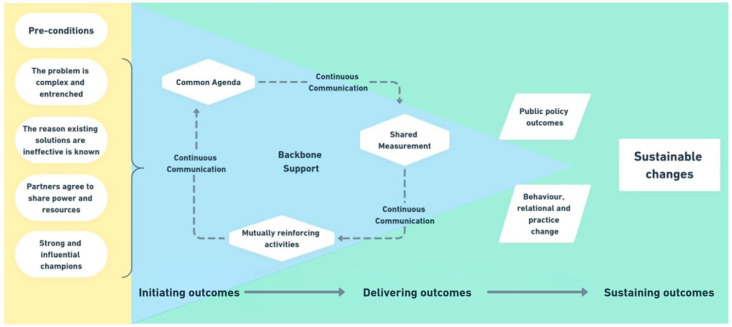
The CHRI model of co-design.

The CHRI model (Figure 1) requires four pre-conditions to be met: the problem is complex and entrenched, the reason that existing solutions are ineffective is known, partners agree to share power and resources, and strong and influential champions are present. The model emphasizes the collective rather than individuals or hierarchies, aligns with respectful engagement and decision-making with Indigenous Elders and communities, enabling significant and measurable improvement to complex health challenges [34]. Adapted from collective impact [35], the CHRI model incorporates five conditions for collective impact: common agenda, backbone support, continuous communication, shared measurement, and mutually reinforcing activities. These conditions support progression through three phases: initiating outcomes, delivering outcomes, and sustaining outcomes. The model ultimately aims to achieve sustainable changes through public policy outcomes and behavior, relational, and practice change [34].

For the PrIDE tool development, the pre-conditions were established through recognition of the complex and entrenched nature of T2DM and CVD in Indigenous Australian communities, understanding the barriers to the existing interventions, an agreement to share power and resources through the CUP governance structure, and leadership from Indigenous investigators and community champions. The tool development phase focused on initiating outcomes through establishing a common agenda for culturally adapted and newly developed tools, providing backbone support through the CHRI infrastructure, maintaining continuous communication through yarning circles, implementing shared measurement through the Aboriginal and Torres Strait Islander Quality Appraisal Tool, and engaging in mutually reinforcing activities across the co-design team. The delivering and sustaining outcomes phases will occur during program implementation and evaluation.

The CHRI model recognizes community contributions in meaningful and significant ways, including opportunities to participate as co-authors on publications and creating local employment opportunities such as Indigenous research assistants [34].

### 2.3. Indigenous Research Methodologies

Yarning circles were used as the primary method for knowledge sharing and co-design. Yarning, an Indigenous research methodology, is a conversational method that involves storytelling and knowledge sharing, emphasizing the establishment of a level platform for knowledge exchange between all participants, recognizing each individual as expert in their own right, and promoting open sharing of experiences and ideas [36]. Between 3 and 5 yarning sessions were conducted for each tool. CUP yarning sessions were held in Bidjigal Country (Sydney, NSW, Australia) or online in 2025.

### 2.4. Ethical Approval

Ethics approval was obtained from the Aboriginal Health and Medical Research Council of NSW (AHMRC) (Approval #2479/25). The study was conducted in accordance with the National Health and Medical Research Council (NHMRC) guidelines for ethical research with Indigenous communities [37].

### 2.5. Tool Development Process

**Figure 2:**
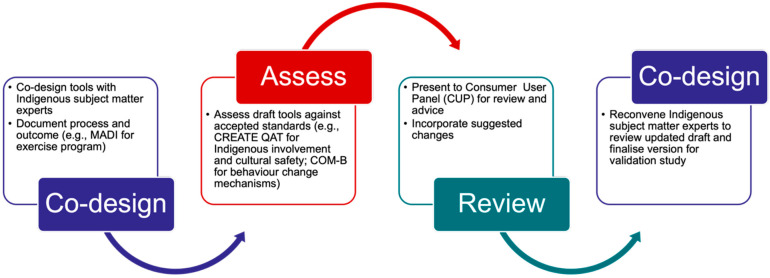
Iterative co-design, assessment, and review cycle used to develop and refine PrIDE tools.

Four tools were developed using the CHRI co-design model in parallel: three culturally adapted tools (the PrIDE Exercise Program, the Strong Spirit Strong Self health self-efficacy assessment tool, and Keep Your Heart Strong educational materials) and one newly developed tool (Success Plan). Both the Strong Spirit Strong Self self-efficacy questionnaire and the Success Plan tool utilize clinical yarning as the delivery method. Clinical yarning is a culturally safe communication approach frequently used in health care, facilitating shared discussion between the research assistant or accredited exercise physiologist and the participant [36,38]. It progresses through interconnected phases: social yarning establishes rapport and trust, diagnostic yarning explores the topic through story-sharing, and management yarning facilitates collaborative planning [36,38]. This structured yet flexible approach is open-ended, story-based, and participant-led, creating space for self-expression and shared understanding. Clinical yarning is culturally centered; shows respect towards Indigenous Australians, privileging Indigenous ways of knowing and communicating; and allows these tools to function as collaborative conversations rather than formal assessments [36,38]. Each tool followed this cycle, with cultural adaptations documented using MADI [39] and all tools reviewed by the CUP for cultural safety and appropriateness. Tool development followed an iterative co-design, assessment, and review cycle, summarized in Figure 2.

### 2.6. PrIDE Exercise Program

The cultural adaptation of the Beat It program was led by an Indigenous accredited exercise physiologist (AEP) from the research team (author BBD), a Bundjalung/Gumbaynggirr (Mid-North Coast region of New South Wales) Indigenous Australian man, with Beat It Program content expertise. The cultural adaptation was systematically documented using the Model for Adaptation Design and Impact (MADI) framework [39], with BBD leading the adaptation process collaboratively with the CHRI co-design team. The completed MADI outlines the specific adaptations made across key program areas including AEP preparation, participant resources, assessment procedures, and program delivery methods. The PrIDE Exercise Program incorporates a combination of aerobic, resistance, balance, and flexibility training, with individualized progression prescribed and supervised by accredited exercise physiologists. Exercise prescription is consistent with contemporary clinical guidelines for adults with T2DM and national physical activity recommendations, including combined aerobic and resistance training and a reduction in sedentary behavior [9,40,41,42].

### 2.7. Strong Spirit Strong Self Health Self-Efficacy Assessment Tool

The 10-item New General Self-Efficacy (NGSE) scale [43] was culturally adapted for assessing health self-efficacy with Indigenous Australians managing T2DM. The adaptation was led by author KC, a Noongar researcher from the Whadjuk clan, in collaboration with author CT, a Whadjuk Noongar Elder. Yarning sessions were conducted with CT, a second Noongar Elder with deep community and cultural knowledge, and an Aboriginal health and community worker with frontline perspectives and expertise from the Champion Centre on Whadjuk/Noongar Country (Perth, WA, Australia).

Multiple yarning sessions allowed for reflection, clarification, and shared understanding to ensure the adapted tool was culturally grounded, practical, and aligned with community expectations and experiences. The adapted tool was renamed “Strong Spirit Strong Self” to reflect its cultural grounding and was subsequently reviewed by the CUP for cultural safety and appropriateness.

### 2.8. Keep Your Heart Strong Educational Materials

**Figure 3:**
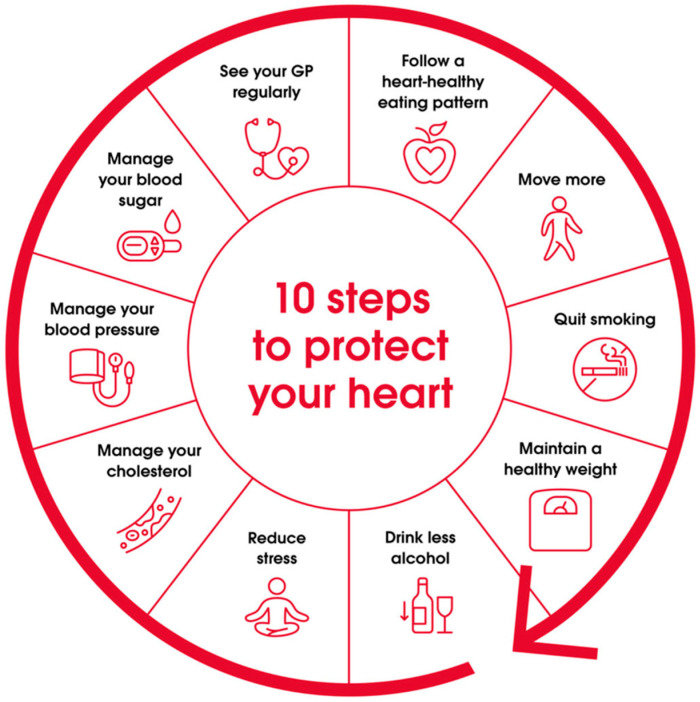
The Australian National Heart Foundation “10 Steps to Protect Your Heart” resource for Indigenous Australians [45].

Heart health educational materials were culturally adapted from the National Heart Foundation’s “10 Steps to Protect Your Heart” resource. The National Heart Foundation is the peak body for heart disease prevention and support in Australia, working to improve heart health outcomes for all Australians through research, advocacy, public awareness campaigns, and accessible resources [44]. The Heart Foundation’s “10 Steps to Protect Your Heart” resource is available on their dedicated Indigenous Australians health webpage [45] (Figure 3 shows the original resource).

The adaptation was led by an Indigenous team member and AEP (author BBD), who reviewed the resource and identified opportunities to enhance accessibility by replacing clinical terminology with everyday language and streamlining content. Working collaboratively with the CHRI co-design team, culturally adapted educational materials were created in two formats: a brochure featuring Markeeta’s (Wemba Wemba) artwork “Paths You Take,” and an animation with voice-over narration by BBD.

### 2.9. The Success Plan

The Success Plan is a newly developed tool designed to facilitate exercise program selection and goal setting in a culturally safe manner. The tool was led and co-developed by an Indigenous AEP (author BBD) in collaboration with the co-design research team. The Success Plan comprises five sequential questions that guide participants through values identification, goal setting, preference assessment, strategy planning, and program selection. The tool reframes traditional pre-exercise questionnaires using simple, strengths-based language that considers the Indigenous Australian context while maintaining important clinical information. The tool was reviewed by the CUP, with feedback leading to refinements in language, tone, and framing, and the inclusion of Elders as role models within the tool to ensure relevance across age groups and cultural contexts.

### 2.10. Assessment of Cultural Appropriateness

All four tools were assessed using the Aboriginal and Torres Strait Islander Quality Appraisal Tool (CREATE) [46] to ensure that they met best practice standards for Indigenous health research. The 14-item CREATE tool assesses domains including community need and consultation, Indigenous leadership and governance, cultural protocols, intellectual property, research paradigm, a strengths-based approach, capacity strengthening, and reciprocal learning opportunities.

### 2.11. Behavior Change Mechanism Analysis

All four tools were analyzed using the COM-B model (Capability, Opportunity, Motivation—Behavior) [47,48,49] to map how tool features align with determinants of behavior change. The COM-B model, a component of the Behavior Change Wheel, posits that behavior occurs when individuals have the capability (physical and psychological), opportunity (social and physical), and motivation (reflective and automatic) to perform it [49]. Each tool component was mapped to the six COM-B domains to assess comprehensive coverage of behavior change mechanisms and to examine how cultural adaptation reconceptualizes these mechanisms in Indigenous contexts. The COM-B analysis presented below reflects an interpretive mapping of behavior change mechanisms as operationalized through co-design in PrIDE, rather than a claim of universal applicability or demonstrated effectiveness.

**Figure 4:**
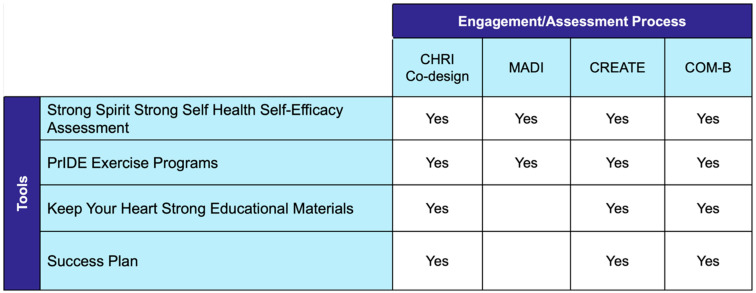
Overview of the engagement and assessment processes applied during PrIDE tool development, indicating which tools underwent co-design, adaptation documentation using the Model for Adaptation Design and Impact (MADI), quality appraisal using the CREATE Quality Appraisal Tool for Indigenous health research, and behavior change analysis using the Capability, Opportunity, Motivation–Behavior (COM-B) model.

Figure 4 summarizes the engagement, adaptation, quality appraisal, and behavior change analysis processes applied to each tool.

### 2.12. Data Management

Tool development processes were documented following principles of Indigenous data sovereignty, with community ownership of knowledge ensuring research outcomes were meaningful and culturally relevant to Indigenous Australian communities and researchers [37].

This paper reports the co-design and development phase only; evaluation of effectiveness, acceptability, and psychometric properties will be undertaken and reported following the implementation of the PrIDE study.

## 3. Results

### 3.1. Overview of Tools Developed

Four tools were successfully developed through the co-design process: three culturally adapted tools (the PrIDE Exercise Program, the Strong Spirit Strong Self health self-efficacy assessment tool, and Keep Your Heart Strong educational materials), and one newly developed tool (Success Plan).

### 3.2. Cultural Appropriateness Assessment

All tools met requirements across key domains of the Aboriginal and Torres Strait Islander Quality Appraisal Tool. Items related to intellectual property agreements and translation to policy/practice and community benefit were marked as “not applicable” as these tools are in the design phase and have not yet been implemented in community settings. Full results are presented in Table 1.

### 3.3. PrIDE Exercise Program Adaptations

The cultural adaptation of the Beat It program preserved core evidence-based mechanisms while enhancing cultural safety, participant engagement, and program accessibility. Adaptations were systematically documented across nine key areas using the MADI framework. The full documentation is presented in Table 2.

### 3.4. Strong Spirit Strong Self Health Self-Efficacy Tool

The cultural adaptation of the NGSE scale preserved core self-efficacy theory while integrating cultural enhancements that reflect Indigenous worldviews and ways of being. The adaptation maintained the 10-item structure to allow for equivalent comprehensiveness and direct comparison with the original tool. Response-scale language was adapted from formal academic terms to everyday language to increase clarity and accessibility. The administration method was adapted from self-administered questionnaire to conversational administration by the research assistant using clinical yarning [36,38].

Content validity was established through yarning with Elders and the community in Nyoongar Country (Perth, WA, Australia). Face validity was established through the co-design process and CUP review. Psychometric validation will be conducted through the PrIDE study, assessing internal consistency reliability, sensitivity to change, convergent validity, known-groups validity, and exploratory factor analysis to examine dimensionality. The full MADI documentation is presented in Table 3.

### 3.5. Keep Your Heart Strong Educational Materials

Heart health educational materials were culturally adapted from the National Heart Foundation’s evidence-based “10 Steps to Protect Your Heart” resource (Figure 3). The adaptation enhanced accessibility by streamlining ten steps to three essential messages delivered through strengths-based framing: (1) make heart-healthy choices, (2) yarn with your clinic mob, and (3) cut back on smokes and alcohol. For the first two messages, framing statements are directly supported by specific, actionable examples provided within the materials to ensure clarity and practical relevance. The adaptation replaced clinical terminology (e.g., “manage your cholesterol”) with everyday language and actionable guidance that resonates with community ways of communicating about health.

**Figure 5:**
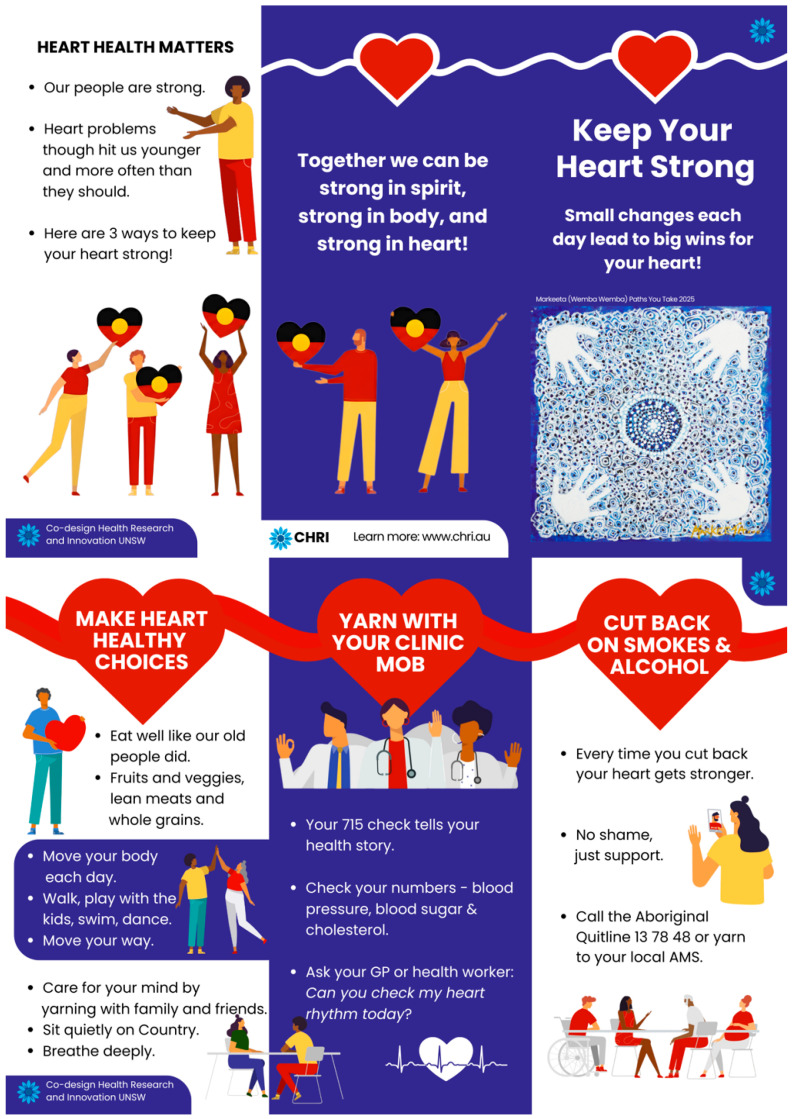
Keep Your Heart Strong three-fold brochure, co-designed for the PrIDE study featuring licensed artwork “Paths You Take” by Markeeta (Wemba Wemba).

The Keep Your Heart Strong brochure and animation feature Markeeta’s artwork “Paths You Take” and include voice-over narration by BBD. CUP feedback was highly positive, with members highlighting the culturally appropriate color palette, integration of Indigenous artwork and visual elements, simplicity of messaging, and use of accessible language. The brochure (Figure 5) will be distributed at program registration and the animation shared on the study’s social media platform.

### 3.6. The Success Plan

The Success Plan integrates multiple theoretical frameworks to support culturally safe, participant-centered decision-making. The tool comprises five sequential questions delivered through clinical yarning. The theoretical rationale for each tool component is presented in Table 4.

### 3.7. Behavior Change Mechanisms: COM-B Analysis

COM-B analysis identified how tool features align with behavior change domains. The PrIDE Exercise Program maps to all six components through its comprehensive adaptations. Strong Spirit Strong Self corresponds to psychological capability and reflective motivation through culturally appropriate assessment that reconceptualizes the capability to include collective, cultural, and spiritual dimensions. Keep Your Heart Strong educational materials align with psychological capability through accessible health information, social and physical opportunity through multiple distribution channels and social connection framing, and both reflective and automatic motivation through strengths-based messaging and culturally resonant visual elements. The Success Plan integrates features that map across all components through a process that corresponds to psychological capability for planning, opportunity assessment through constraint identification and strategy planning, and reflective and automatic motivation. The complete COM-B mapping for all four PrIDE tools is presented in Supplementary Table S1.

### 3.8. CUP Feedback and Iterative Refinement

The CUP provided substantive feedback across all four tools, resulting in 1–3 rounds of revision for each tool. Primary improvements included adjustments to language, tone, and framing to ensure accessibility, cultural appropriateness, and respectful communication across age groups and literacy levels. For example, a CUP review of the Success Plan led to the addition of “Be a role-model for my community” as a program goal option; the removal of “I will” prefixes from barrier-planning options, as CUP members identified this phrasing as pressuring rather than supportive; and the substitution of “family” with “mob” to better reflect community language and collective responsibility. Elder perspectives were integrated throughout tools to reflect intergenerational knowledge and support structures. The CUP provided guidance on integration of licensed artwork throughout materials and refinement of delivery methods to ensure cultural safety and participant comfort.

## 4. Discussion

### 4.1. Principal Findings

This study developed four tools for the Preventing Indigenous CVD and Diabetes through Exercise (PrIDE) study [33] using co-design following best practice frameworks: three culturally adapted tools (the PrIDE Exercise Program from Beat It, the Strong Spirit Strong Self from NGSE, the Keep Your Heart Strong from Heart Foundation resources) and one newly developed tool (the Success Plan). All tools met Indigenous health research quality standards via the Aboriginal and Torres Strait Islander Quality Appraisal Tool [46], were governed through Indigenous leadership structures (CAG and CUP), and utilized Indigenous research methodologies, primarily yarning [36].

Indigenous leadership throughout the development process ensured that cultural knowledges and Indigenous epistemologies shaped tool design from inception rather than through consultation. This approach aligns with emerging diabetes co-design initiatives in Australia that privilege Indigenous governance and lived experience [30,31], and extends them by embedding structured adaptation and theory mapping within an exercise intervention context.

MADI documentation provides transparent, prospective recording of adaptation decisions while preserving core evidence-based mechanisms and enhancing cultural safety, addressing a gap where cultural adaptations are often reported superficially, limiting replication. COM-B analysis revealed how co-design reconceptualized the operationalization of behavior change mechanisms: psychological capability was conceptualized to include collective, cultural, and spiritual dimensions; social opportunity was understood as requiring deliberate creation through Indigenous leadership and trusted relationships; and motivation was operationalized holistically and relationally. The evaluation of effectiveness, acceptability, and scalability will be reported following PrIDE implementation.

### 4.2. Comparison with Prior Work

The published evidence on culturally adapted exercise interventions for Indigenous Australians with T2DM remains limited [15]. Previous reviews have noted not only the paucity of Indigenous exercise interventions, but also the limited reporting of development processes and Indigenous governance, constraining reproducibility and translation to other communities [18]. International evidence is sparse; one supervised community-based group exercise intervention with Indigenous Polynesian adults demonstrated improvements in quality of life and cardiometabolic outcomes [16,17]. However, this evidence focused primarily on outcome evaluation and provided limited detail on program development processes, governance structures, or systematic cultural adaptation approaches, constraining reproducibility and transferability to other Indigenous populations. PrIDE extends this evidence base by pairing an established, evidence-based exercise program with Indigenous governance and explicitly documenting cultural adaptation using structured frameworks, responding to calls for greater transparency, Indigenous leadership, and methodological rigor in co-designed health interventions [34]. While specific cultural adaptations are necessarily locally determined, the process framework—combining Indigenous-governed co-design (CHRI model), structured adaptation documentation (MADI), quality appraisal (CREATE), and theory mapping (COM-B)—is transferable to exercise intervention development with Indigenous peoples in other high-income colonized countries. Critically, this study positions cultural adaptation processes and theory-informed design decisions as primary objects of inquiry rather than implicit background activity.

### 4.3. Tool-Specific Contributions

Strong Spirit Strong Self addresses a recognized gap in Indigenous health research regarding culturally appropriate measurement. Western self-efficacy instruments privilege individualistic constructs that may be misaligned with Indigenous worldviews [7,66]. While Bandura [67] acknowledged that efficacy beliefs operate through both personal and collective agency, standard self-efficacy questionnaires typically operationalize the construct within individualistic frameworks. Yet self-efficacy—belief in one’s capability to take action—is strengthened, supported, and sustained through relationships and social context for all people [68]. For Indigenous Australians, this relational foundation is particularly salient, with family, Elders, community, and culture recognized as essential sources of strength that lift and sustain the individual [7]. Strong Spirit Strong Self preserves the theoretical core of self-efficacy while reconceptualizing its sources to include family, Elders, culture, spirit, and community, reflecting relational and collective dimensions of capability.

The Success Plan demonstrates how traditional intake processes can be reframed through strengths-based language and clinical yarning for culturally safe decision-making while maintaining clinical utility. By positioning barrier navigation as proactive coping drawing on collective resources, it integrates behavioral theory with Indigenous relational values.

Keep Your Heart Strong illustrates that even resources targeting Indigenous audiences benefit from Indigenous-led adaptation. Indigenous review identified barriers in clinical language, framing, and information density. Streamlining content and reframing through strengths-based language enhanced the accessibility while maintaining evidence-based content.

### 4.4. Co-Design and Behavior Change Mechanisms

COM-B analysis revealed how co-design reshaped the operationalization and interpretation of behavior change mechanisms. Psychological capability was conceptualized to include collective efficacy, cultural knowledge, and spiritual strength, positioning family, Elders, and community as intrinsic capability resources rather than external supports—challenging individualistic assumptions in Western self-efficacy models [7,66].

Social opportunity was conceptualized as a prerequisite for engagement rather than optional enhancement. Historical trauma, ongoing racism, and institutional mistrust necessitate deliberate creation of social opportunity through Indigenous leadership, culturally congruent communication, and trusted intermediaries [34]. Analysis revealed that PrIDE incorporated features mapping to social opportunity through governance structures, recruitment strategies, and delivery approaches.

Motivation was conceptualized holistically and relationally, with goals anchored in family and community responsibilities rather than solely individual outcomes, extending autonomy-focused theories to include culturally congruent autonomy grounded in interdependence [51]. COM-B mapping identified that Keep Your Heart Strong’s integration of Indigenous artwork, voice-over, and relational framing (“yarn with your clinic mob”) aligns with automatic motivation through cultural resonance while streamlined content corresponds to psychological capability.

These findings demonstrate how co-design informs application of established behavior change frameworks in culturally congruent ways, advancing the understanding of theory operationalization in Indigenous health contexts.

### 4.5. Strengths and Limitations

A key strength of this work is the use of structured co-design and adaptation frameworks alongside Indigenous governance and research methodologies. The integration of the CHRI model, MADI framework, and Aboriginal and Torres Strait Islander Quality Appraisal Tool provided methodological rigor while centering Indigenous leadership and accountability.

Several limitations should be noted. Psychometric validation of Strong Spirit Strong Self is pending and will be conducted during PrIDE implementation. Tool effectiveness and acceptability have not yet been evaluated and will be reported separately. Tool development occurred with specific communities and may not generalize across the diversity of Indigenous Australian nations, languages, and cultural practices; further adaptation may be required in new contexts.

### 4.6. Implications for Practice and Research

Health services can use PrIDE as a model for implementing exercise interventions for Indigenous Australians with T2DM, with MADI documentation providing detailed adaptation guidance. Strong Spirit Strong Self provides a culturally appropriate self-efficacy instrument for Indigenous populations; researchers should prioritize culturally adapted outcome measures over standard Western instruments. The Success Plan demonstrates clinical yarning integration into routine practice for culturally safe assessment and shared decision-making, applicable beyond exercise interventions. Keep Your Heart Strong demonstrates value in review of existing resources to enhance accessibility. Sustainability has been considered within the design of the PrIDE tools through alignment with routine accredited exercise physiologist practice and existing service workflows, supporting ongoing use beyond the research phase.

For researchers, this body of work demonstrates feasibility of systematic cultural adaptation using established frameworks. The CHRI model and MADI framework combination provides methodological rigor while centering Indigenous knowledge, leadership, and community control. Indigenous-governed work ensures cultural authenticity unachievable through consultation-based approaches. Future research should prioritize Indigenous leadership in systematic adaptations for chronic disease prevention and management interventions.

Implementation in remote and rural communities will provide evidence on effectiveness, acceptability, and scalability. Evaluation will examine improvements in cardiovascular risk factors, physical fitness, and self-efficacy, and whether improvements are sustained. Process evaluation will identify implementation facilitators and barriers, informing scale-up planning and policy.

## 5. Conclusions

This research demonstrates systematic adaptation and development of tools for Indigenous health through Indigenous-governed co-design processes honoring Indigenous knowledge systems while maintaining evidence-based mechanisms. The three culturally adapted tools and newly developed Success Plan address evidence gaps in interventions with Indigenous Australians. MADI documentation and theoretical rationale provide a model for future cultural adaptation and development of research. PrIDE study implementation will provide evidence on effectiveness, acceptability, and scalability to inform practice, policy, and research aimed at improving health equity with Indigenous Australians.

## Figures and Tables

**Table 1:** Aboriginal and Torres Strait Islander Quality Appraisal Tool (CREATE) assessment results for PrIDE Tools.

Aboriginal and Torres Strait Islander Quality Appraisal Tool (Yes, Partially, No, Unclear)	PrIDE Exercise Program	Strong Spirit Strong Self Self-Efficacy Assessment	Keep Your Heart Strong Educational Materials	Success Plan
Did the research respond to a need or priority determined by the community?	Yes	Yes	Yes	Yes
Was community consultation and engagement appropriately inclusive?	Yes	Yes	Yes	Yes
Did the research have Aboriginal and Torres Strait Islander research leadership?	Yes	Yes	Yes	Yes
Did the research have Aboriginal and Torres Strait Islander governance?	Yes	Yes	Yes	Yes
Were local community protocols respected and followed?	Yes	Yes	Yes	Yes
Did the researchers negotiate agreements in regard to rights of access to Aboriginal and Torres Strait Islander peoples’ existing intellectual and cultural property?	Not applicable	Not applicable	Not applicable	Not applicable
Did the researchers negotiate agreements to protect Aboriginal and Torres Strait Islander peoples’ ownership of intellectual and cultural property created through the research?	Not applicable	Not applicable	Not applicable	Not applicable
Did Aboriginal and Torres Strait Islander peoples and communities have control over the collection and management of research materials?	Not applicable	Not applicable	Not applicable	Not applicable
Was the research guided by an Indigenous research paradigm?	Yes	Yes	Yes	Yes
Does the research take a strengths-based approach, acknowledging and moving beyond practices that have harmed Aboriginal and Torres Strait peoples in the past?	Yes	Yes	Yes	Yes
Did the researchers plan and translate the findings into sustainable changes in policy and/or practice?	Not applicable	Not applicable	Not applicable	Not applicable
Did the research benefit the participants and Aboriginal and Torres Strait Islander communities?	Not applicable	Not applicable	Not applicable	Not applicable
Did the research demonstrate capacity strengthening for Aboriginal and Torres Strait Islander individuals?	Yes	Yes	Yes	Yes
Did everyone involved in the research have opportunities to learn from each other?	Yes	Yes	Yes	Yes

**Table 2:** Model for Adaptation Design and Impact (MADI)—initial cultural adaptation of Beat It program.

Adaptation Areas	Beat It (In-Person)	PrIDE (In-Person)	Rationale for Adaptation	Core Mechanism Impact
Accredited Exercise Physiologist training	- 12 h web-based learning and 1-day in-person practical training	- Additional 1 h web-based learning on the PriDE research study protocol, facilitator requirements and key considerations. Topics also include conducting ethical research in Indigenous populations, and the CHRI Model of Co-design - Additional 5 h web-based learning about Indigenous Australians and cultural respect in action. Topics include respect and reciprocity, cultural protocols, strengths-based language, colonization, white privilege, cultural load, racism, equity, anti-racist skill set, social and cultural determinants of health, cultural responsiveness, cultural humility, cultural safety in health settings, clinical yarning and self-reflexivity - Cultural supervision of AEPs during intervention	Ensures the AEP is equipped with the knowledge and confidence to conduct themselves and their delivery style in a culturally safe, ethical and effective manner. The additional training facilitates the AEP’s capability to build trust, communicate in a strengths-based format, and respond to their participants’ unique cultural protocols while maintaining Beat It’s evidence-based approach.	Preserved: Beat It core training content Enhanced: cultural safety, cultural humility, participant and AEP trust, AEP cultural protocol confidence (availability of supervision to guide confidence)
Marketing	- Direct mail (via post), email, and website	- Recruitment through direct mail, (via post), phone calls, email, website and social media targeting Aboriginal Community Controlled Health Organizations (ACCHOs) and Aboriginal Medical Services (AMS).	Recruitment through trusted, Aboriginal-owned and governed organizations (ACCHOs/AMS) enhances program reach and credibility, while helping to navigate barriers created by intergenerational trauma and historical experiences with government-run institutions.	Preserved: original marketing remains part of strategy Enhanced: exposure and buy-in from community are strengthened through trusted organizations marketing approach
Participant resources	- Beat It participant handbook, home exercise resource - Theraband	- All participant-facing resources co-designed and include licensed Indigenous artwork and culturally safe visual elements throughout - PrIDE participant handout - Education session topics determined by each community site - Success Plan: co-designed, Indigenous AEP-led tool for personalized exercise strategy selection and goal setting - TGA-approved Withings ScanWatch 2 and Withings smartphone application - Theraband	Co-designed resources with Indigenous artwork build sense of community ownership in the program which facilitates participant self-determination. The Success Plan reframes pre-exercise questionnaire with simple, strengths-based language that considers the Indigenous Australian context while not omitting important information. Withings ScanWatch and accompanying smartphone application support education and intrinsic motivation with real-time feedback.	Preserved: exercise equipment resources. Participant handbook and home exercise resources remain Enhanced: intrinsic motivation, community buy-in via co-design of culturally important materials, strengths-based, culturally safe resources, technology-driven feedback for increased engagement and education
Trainer resources	- Beat It in-person delivery manual and Beat It facilitator manual (educational sessions)	- PrIDE delivery and facilitator manual to include study protocol procedures and processes - RA training materials for setting up Withings ScanWatch 2 and Withings smartphone application	RA training materials support consistent participant engagement with wearable technology, while the PrIDE manual ensures standardized and reliable delivery of study procedures.	Preserved: standardized procedures maintain a consistent approach for both AEP and RA Enhanced: Withings ScanWatch integration with structured support manual
Medical clearance	- Standardized medical clearance form, including recommended program inclusion or exclusion criteria, medical history, medications, and latest hemoglobin A1c and lipid test results; participants typically bring a physical copy of the medical clearance form to initial consultation with a Beat It trainer	- Standardized medical clearance from general practitioner required	Simplifies process while maintaining the safety of participants, reduces extra friction that may act as a barrier to participation (access to printer to bring physical copy of clearance form). Maintains continuity of care with trusted local GP.	Preserved: pre-engagement safety mechanism via qualified health professional Enhanced: reduced friction that considers common barriers for Indigenous Australian peoples (socio-economic disadvantage, patient–doctor mistrust)
Preprogram	- Preprogram resources sent including welcome letter confirming program registration, medical clearance, and initial consultation process and the Beat It trainer books initial assessment appointment	- Preprogram phone call by AEP/RA to each participant confirming PrIDE study registration	Preprogram call designed to build rapport, confirm registration, intent to attend and address barriers to engagement (transport, scheduling, pre-conceptions).	Preserved: AEP books initial assessment Enhanced: rapport building, common barriers addressed in phone call to improve adherence and attendance
Initial and final assessment	- Conducted in person - Obtain medical clearance, participant informed consent, and emergency contact information and complete prescreening questionnaire - Complete baseline measurements, including height, weight, waist circumference, blood pressure, and resting heart rate - Complete exercise tests including the 6 min walk test, 30 s sit-to-stand test, 30 s arm curl test, seated sit-and-reach test, and one-legged stand test - Goal setting	- Program registration event in-person for all participants - Obtain medical clearance and participant informed consent - Provision of program resources in-person - Complete pre- and post-evaluation data including demographics, culturally adapted 8-item New General Self-Efficacy (NGSE) tool for health self-efficacy assessment - Assessments conducted by accredited exercise physiologist (AEP) with RA present - Complete baseline measurements, including height, weight, waist circumference, blood pressure, and resting heart rate - Complete exercise tests including the 6 min walk test, 30 s sit-to-stand test, 30 s arm curl test, seated sit-and-reach test, and one-legged stand test - Success Plan completed collaboratively with AEP to facilitate personalized exercise strategy selection (group program or individual program) and goal setting	Incorporating an Indigenous RA fosters a culturally safe environment, helping to reduce anxiety around medical assessments in the context of the intergenerational trauma experienced by Indigenous Australians. Using the adapted NGSE alongside the Success Plan emphasizes a culturally informed approach to personalized goal setting for each participant.	Preserved: exercise assessment measures and informed consent Enhanced: culturally safe assessment environment and adapted self-efficacy questionnaire and Success Plan designed with the Indigenous Australian context considered
Exercise sessions	- Capped at 12 participants per session - In-person group exercise sessions consist of a warm-up, followed by a combination of aerobic, resistance, balance, and flexibility exercises tailored to participants abilities, followed by a cooldown period	- Participants select either group exercise sessions or individual exercise sessions based on personal preference and circumstances - Capped at 12 participants per session - AEPs encouraged to factor in rapport building into structure of sessions, e.g., group activities, ‘pairing’ participants together, promotion of conversation, and relationship building - Exercise prescription, supervision and progression individualized by AEP - Exercise program culturally adapted by Indigenous AEP	Encouraging rapport building within exercise sessions reflects Indigenous ways of being (yarning), enhancing participant comfort, satisfaction, and adherence. Offering group or individual options helps navigate common barriers faced by Indigenous Australians, such as transport, family responsibilities, and community connection. The exercise selection is adapted to maintain evidence-based prescription principles while also reflecting Indigenous Australians’ holistic perspective on health and wellbeing.	Preserved: exercise physiology principles, exercise progression/regression, AEP supervision, minimum effective dosage, exercise selection, gradual progressive overload Enhanced: social support, exercise delivery format choice, rapport building strategies inbuilt, culturally appropriate exercise selection to align with Indigenous Australian way of being/holistic health perspective
Education sessions	- 6 × 30 min person-centered education sessions on various lifestyle and diabetes management topics delivered in person	- All participants have access to a Facebook group where short education messages, prompts, and video resources are posted - Group participants: education integrated into conversations throughout exercise sessions - Individual participants: education delivered through weekly in-person check-ins with AEP, text conversations, or phone calls - Education topics may include using wearable technology to monitor health, self-management of T2DM, managing energy, and moving well - Fortnightly ‘cook ups’ where all participants gather to share a meal with AEP demonstrating healthy meal preparation (aligning with Australian Guidelines for Healthy Eating) using locally available foods and allowing for participant-directed yarning about health and nutrition topics	Shifting the education strategy into an integrated model including group conversations, social media groups and gatherings for “cook ups” aligns with Indigenous Australian community practices (yarning, community gatherings), making the educational session feel more informal, relevant, and co-delivered without omitting any vital information.	Preserved: evidence-based educational content on various lifestyle and diabetes management topics Enhanced: co-owned education sessions, yarning circles and community gatherings, social media platforms to facilitate ongoing connection with peers and AEP

Note: AEP = accredited exercise physiologist; CHRI = Co-design Health Research and Innovation; CVD = cardiovascular disease; NGSE = New General Self-Efficacy; RA = research assistant; T2DM = Type 2 diabetes mellitus; TGA = Therapeutic Goods Administration.

**Table 3:** Model for Adaptation Design and Impact (MADI)—Strong Spirit Strong Self (self-efficacy assessment tool).

MADI Domain	Original: General Self-Efficacy Scale (GSE)	Adapted: Strong Spirit Strong Self	Rationale for Adaptation	Core Mechanism Impact
Content/Concept	Western individualistic self-efficacy construct; focus on personal resourcefulness and independent problem-solving	Self-efficacy reconceptualized to include collective efficacy; spirit, culture, and community as sources of strength; interdependence valued alongside independence	Indigenous worldviews center interconnectedness; self exists in relation to family, community, culture; help-seeking is strength and wisdom	Preserved: Self-efficacy as belief in capability to achieve goals. Enhanced: Added cultural and collective dimensions make construct more valid and meaningful for Indigenous populations
Number of Items	10 items	10 items	Maintained same number of items for equivalent comprehensiveness; one-to-one adaptation allows for direct comparison	Preserved: Assessment comprehensiveness and breadth
Response Scale	4-point: Not at all true, Hardly true, Moderately true, Exactly true	4-point: Not at all true, A little true, Mostly true, Very true	Everyday language (a little, mostly, very) more familiar and comfortable than formal terms (hardly, moderately, exactly); increases clarity	Preserved: 4-point Likert structure maintains sensitivity. Enhanced: clearer response options support accurate responding
Item 1: Perseverance	I can always manage to solve difficult problems if I try hard enough	When things get hard, I can keep trying until I find a way.	Removed “absolute/always” to reflect realistic experiences; “things get hard” acknowledges lived reality; process-oriented “keep trying” emphasizes persistence as ongoing strength	Preserved: persistence in face of difficulty. Modified: More realistic framing validates lived experience
Item 2: Assertion	If someone opposes me, I can find the means and ways to get what I want.	If people don’t agree with me, I can still stand strong and get what I need in a good way.	“Don’t agree” reflects respectful disagreement rather than opposition; “what I need” validates legitimate needs; in a good way, centers Indigenous value of maintaining relationships and harmony while asserting needs	Preserved: ability to advocate for self. Enhanced: cultural value of maintaining relationships while asserting needs; respectful assertion as strength
Item 3: Goal pursuit	It is easy for me to stick to my aims and accomplish my goals.	I can stay on track with my goals, even when life gets tough.	Removed “easy to acknowledge that goals require real effort”; added “even when life gets tough”, which validates challenges while affirming capability; “stay on track” reflects ongoing commitment	Preserved: goal-directed behavior. Enhanced: acknowledges real-world challenges while affirming capability and persistence
Item 4: Unexpected events	I am confident that I could deal efficiently with unexpected events.	I feel sure I can handle things that come up without warning.	“Feel sure” reflects embodied knowing and confidence; removed efficiency emphasis to focus on capability itself; “handle” reflects practical capability	Preserved: confidence in managing uncertainty. Modified: emphasis on capability rather than efficiency; values embodied confidence
Item 5: Resourcefulness	Thanks to my resourcefulness, I know how to handle unforeseen situations.	I know how to deal with new things, using my own skills or asking family, Elders, or community for help.	Explicitly includes collective support and help-seeking as resourceful strategies; Elders valued as sources of wisdom and guidance; reflects Indigenous value of interdependence	Preserved: ability to handle novel situations. Enhanced: interdependence as resource and strength; recognizes collective wisdom; reflects cultural values of connection
Item 6: Problem-solving effort	I can solve most problems if I invest the necessary effort.	If I put in the effort, I can sort most problems out.	Everyday language (“put in the effort”, “sort out”) more accessible and conversational; maintains core message about effort and capability	Preserved: effort–outcome relationship. Enhanced: more accessible, conversational language supports engagement
Item 7: Coping under stress	I can remain calm when facing difficulties because I can rely on my coping abilities.	When times are hard, I can stay calm by leaning on my spirit, culture, and what I’ve learnt.	Spirit and culture explicitly named as sources of strength and resilience; “leaning on” acknowledges support systems and connections; includes both traditional wisdom and contemporary knowledge	Preserved: emotional regulation under stress. Enhanced: cultural and spiritual dimensions of strength and resilience; wholistic health and multiple sources of support
Item 8: Multiple solutions	When I am confronted with a problem, I can usually find several solutions.	When a problem comes up, I can usually think of a few different ways to handle it.	“Comes up” replaced confronted for more natural phrasing; everyday language (“a few different ways”) more conversational; maintains core concept of flexible thinking	Preserved: flexible problem-solving. Enhanced: natural, conversational framing supports comfortable engagement
Item 9: Trouble/solutions	If I am in trouble, I can usually think of a solution	If I get into trouble, I can find a way to make things right, maybe with guidance from family or Elders.	“Make things right” emphasizes relational repair and restoration; guidance from family/Elders explicitly valued as wisdom and support; reflects restorative approaches and cultural value of seeking guidance	Preserved: problem-solving capability. Enhanced: relational dimension and restoration; values help-seeking and Elder wisdom as strengths; reflects cultural values of connection and guidance
Item 10: General capability	I can usually handle whatever comes my way.	Whatever life puts in front of me, I believe I can handle it, especially with culture and community around me.	Culture and community explicitly recognized as essential sources of strength; “I believe” acknowledges faith and hope; “especially with” centers cultural connection and community support as foundations of capability	Preserved: general self-efficacy. Enhanced: collective efficacy and cultural connection as sources of strength; interdependence and community as foundations
Administration	Self-administered questionnaire	Administered by research assistant (RA); questionnaire completed together in conversational tone; participant can ask for clarification at any time	Completing questionnaire together with RA creates space for reflection and discussion; enables participants to share their interpretation and meaning making; builds relationship and rapport; reflects oral communication traditions; ensures that questions are understood as intended; accommodates diverse literacy levels; collaborative approach respects participant as expert in their own experience	Preserved: standardized assessment. Enhanced: culturally safe, collaborative administration; relational approach; participant agency and voice centered; respects oral traditions; accessible across literacy levels
Validation	Extensively validated in Western populations; high reliability (α = 0.75–0.91)	Content validity established through yarning with Elders and community; face validity through co-design process; psychometric validation to be conducted through the PrIDE study	Cultural adaptation creates a new tool requiring validation with Indigenous populations; content validity established through Elder wisdom and community knowledge, which ensures cultural appropriateness; face validity through co-design ensures that tool is meaningful and acceptable; psychometric properties will be assessed in PrIDE study	Psychometric properties to be assessed through PrIDE study: internal consistency reliability (Cronbach’s α); sensitivity to change (pre-post intervention); convergent validity (correlations with program adherence and physical activity); known-groups validity (program completers vs. non-completers); exploratory factor analysis used to examine dimensionality (unidimensional vs. individual and collective efficacy dimensions)

**Table 4:** PrIDE Success Plan—theoretical rationale.

Tool Component	Theoretical Framework and Rationale
Tool Delivery Method	Clinical Yarning [36] - The Success Plan is administered through clinical yarning, a culturally safe communication approach that facilitates conversation between the AEP and the participant [38] - Yarning is an Indigenous Australian conversational method that is open-ended, story-based, and participant-led, creating space for self-expression and shared understanding [36] - This delivery method privileges Indigenous ways of knowing and communicating, supporting rapport-building and trust [36] - Clinical yarning allows the tool to function as a collaborative conversation rather than a formal assessment, aligning with principles of shared decision-making and person-centered care [38]
Q1: What are you hoping to get from this program? (You can tick more than one) Options: Have more energy; Feel stronger; Feel happier; Feel better; Feel relaxed; Lose weight; Look after my diabetes; Be around longer for my family; Be a role-model for my community; Look after my heart; Learn exercises I can keep doing; Meet like-minded people; Learn about healthy eating; Other	Self-Determination Theory (SDT)—Intrinsic Motivation - Identifies participants’ intrinsic motivations and personal values - SDT shows that when people exercise for autonomous reasons (personal values) rather than controlled reasons (external pressure), they have better adherence and outcomes [50,51] - Allowing multiple selections acknowledges that health goals are interconnected, not singular [50,51] Holistic Health Models [7,52] - Options span physical (stronger, energy), emotional (happier, relaxed), social (family, meet people), and functional (learn exercises) domains - “Be around longer for my family” recognizes relational health motivators - “Be a role-model for my community” recognizes identity-based and collective health motivators
Q2: In 3 months what would you feel proud of achieving? (Open-ended)	Goal-Setting Theory - Specific, time-bound goals improve adherence and outcomes [53] - Self-articulated goals are more meaningful than clinician-imposed goals [54] - The 3-month timeframe extends beyond the 8-week program, encouraging participants to envision sustainable change and continued progress after program completion [55] Strengths-Based Approach - Asking about “pride” and “achievement” frames goals positively around capabilities and aspirations rather than deficits or problems [56] - These shifts focus from what’s wrong (symptom management, disease control) to what’s possible (personal accomplishments, meaningful outcomes) [56] - Open-ended format aligns with yarning methodology, allowing participants to express goals in their own words beyond clinical measures (e.g., family participation, cultural activities, functional abilities) [36,38] - Creates psychological ownership of outcomes [51]
Q3: When you think about exercise, what sounds better to you? Options: Exercising in a group at the same time each week with an instructor; Exercising on my own with a plan and regular check ins; I’m happy with either	Person-Centered Care - Assesses individual preference for learning/activity style [51] - Acknowledges different people thrive in different contexts [57] Self-Determination Theory (Autonomy) - Providing choice supports autonomy, a key driver of intrinsic motivation [50,51] - “I’m happy with either” prevents forced choice - Frames positively (“what sounds better”) rather than focusing on barriers first [58]
Q4: How will you balance being in this program and other activities? Options: Ask mob for help with the kids/grannies; Ask my neighbor to drive me; Use my lunch break; Arrange things around my other activities; Give it a go even if I don’t feel like it; Reach out to a friend for encouragement; Show up; Other	Implementation Intentions Theory - Pre-planning how to overcome obstacles improves adherence [55,59] - “If–then” planning bridges intention–behavior gap [55,59] Proactive Coping - Shifts from identifying barriers to identifying solutions/strategies [60] - Options span practical (childcare, transport), temporal (lunch break, scheduling), motivational (give it a go), and social (friend support) strategies [60] - Positions participants as capable problem-solvers, not passive recipients [56,61]
Q5: Which program do you think would work best for you Options: Group program—exercise with others 2 times per week for 8 weeks. Meet face-to-face with support/individual program—exercise on your own with a plan made with you. Regular check-ins by phone, text, or visit	Shared Decision-Making - Final decision point after reflection on goals, preferences, and circumstances [62,63] - Placing this last ensures the decision is informed, not arbitrary [37] Self-Determination Theory (Autonomy) - Honors participant self-determination and agency [51] - Clear, accessible descriptions of key distinguishing features without judgment about either choice [58] - Recognizes both programs as valid, effective options [58]
Overall Tool Design	Universal Design for Learning (UDL) [64] - Multiple means of expression (checkboxes, open-ended, choices) - Builds sequentially: values → goals → preferences → strategies → decision Plain Language and Health Literacy - Accessible language throughout [65] - Strengths-based framing (no deficit language) [56]

## Data Availability

All data generated during this methods paper are included in this article. Qualitative data from co-design yarning sessions are not publicly available due to Indigenous data sovereignty principles and participant privacy protections.

## Supplementary Materials

The following supporting information can be downloaded at https://www.mdpi.com/article/10.3390/ijerph23020252/s1, Table S1: COM-B Analysis: How PrIDE Tools Address Behavior Change Mechanisms.

## Abbreviations

The following abbreviations are used in this manuscript:

AEP	Accredited exercise physiologist
ACCHO	Aboriginal Community-Controlled Health Organization
AHMRC	Aboriginal Health and Medical Research Council
AMS	Aboriginal Medical Service
CAG	Consumer Advisory Group
CHRI	Co-design Health Research and Innovation
COM-B	Capability, Opportunity, Motivation—Behavior (model)
CREATE	Aboriginal and Torres Strait Islander Quality Appraisal Tool
CUP	Consumer User Panel
CVD	Cardiovascular disease
MADI	Model for Adaptation Design and Impact
NGSE	New General Self-Efficacy (scale)
NHMRC	National Health and Medical Research Council
NSW	New South Wales
PrIDE	Preventing Indigenous Cardiovascular Disease and Diabetes through Exercise
RA	Research assistant
SDT	Self-Determination Theory
T2DM	Type 2 diabetes mellitus
TGA	Therapeutic Goods Administration
UDL	Universal Design for Learning

## References

[1] Brown A., Carrington M.J., McGrady M., Lee G., Zeitz C., Krum H., Rowley K., Stewart S. (2014). Cardiometabolic Risk and Disease in Indigenous Australians: The Heart of the Heart Study. Int. J. Cardiol..

[2] Australian Institute of Health and Welfare (2024). Aboriginal and Torres Strait Islander Health Performance Framework—Summary Report.

[3] Australian Institute of Health and Welfare (2022). Australian Burden of Disease Study: Impact and Causes of Illness and Death in Aboriginal and Torres Strait Islander People 2018.

[4] Australian Institute of Health and Welfare Rural and Remote Health. https://www.aihw.gov.au/reports/rural-remote-australians/rural-and-remote-health.

[5] Brown A., Kritharides L. (2017). Overcoming Cardiovascular Disease in Indigenous Australians. Med. J. Aust..

[6] Bainbridge R., McCalman J., Clifford A., Tsey K. (2015). Cultural Competency in the Delivery of Health Services for Indigenous People.

[7] Dudgeon P., Harris J., Newnham K., Brideson T., Cranney J., Darlaston-Jones D., Hammond S.W., Herbert H., Homewood J., Page S., Dudgeon P., Milroy H., Walker R. (2014). Working Together: Aboriginal and Torres Strait Islander Mental Health and Wellbeing Principles and Practice.

[8] De Zilva S., Walker T., Palermo C., Brimblecombe J. (2022). Culturally Safe Health Care Practice for Indigenous Peoples in Australia: A Systematic Meta-Ethnographic Review. J. Health Serv. Res. Policy.

[9] American Diabetes Association Professional Practice Committee (2024). Introduction and Methodology: Standards of Care in Diabetes—2024. Diabetes Care.

[10] Umpierre D., Ribeiro P.A.B., Kramer C.K., Leitão C.B., Zucatti A.T.N., Azevedo M.J., Gross J.L., Ribeiro J.P., Schaan B.D. (2011). Physical Activity Advice Only or Structured Exercise Training and Association With HbA1c Levels in Type 2 Diabetes: A Systematic Review and Meta-Analysis. JAMA.

[11] White L., Kirwan M., Christie V., Hurst L., Gwynne K. (2024). The Effectiveness of Clinician-Led Community-Based Group Exercise Interventions on Health Outcomes in Adults with Type 2 Diabetes Mellitus: A Systematic Review and Meta-Analysis. Int. J. Environ. Res. Public Health.

[12] Gracey M., King M. (2009). Indigenous Health Part 1: Determinants and Disease Patterns. Lancet.

[13] Marmot M., Friel S., Bell R., Houweling T.A., Taylor S., on behalf of the Commission on Social Determinants of Health (2008). Closing the Gap in a Generation: Health Equity through Action on the Social Determinants of Health. Lancet.

[14] Vincze L., Barnes K., Somerville M., Littlewood R., Atkins H., Rogany A., Williams L.T. (2021). Cultural Adaptation of Health Interventions Including a Nutrition Component in Indigenous Peoples: A Systematic Scoping Review. Int. J. Equity Health.

[15] Hurst L., Kirwan M., Christie V., Cross C., Baylis S., White L., Gwynne K. (2024). The Effect of Community-Based Exercise on Health Outcomes for Indigenous Peoples with Type 2 Diabetes: A Systematic Review. Int. J. Environ. Res. Public Health.

[16] Sukala W.R., Page R., Lonsdale C., Lys I., Rowlands D., Krebs J., Leikis M., Cheema B.S. (2013). Exercise Improves Quality of Life in Indigenous Polynesian Peoples With Type 2 Diabetes and Visceral Obesity. J. Phys. Act. Health.

[17] Sukala W.R., Page R., Rowlands D.S., Krebs J., Lys I., Leikis M., Pearce J., Cheema B.S. (2012). South Pacific Islanders Resist Type 2 Diabetes: Comparison of Aerobic and Resistance Training. Eur. J. Appl. Physiol..

[18] Meharg D.P., Gwynne K., Gilroy J., Alison J.A. (2020). Exercise-Based Interventions for Indigenous Adults with Chronic Lung Disease in Australia, Canada, New Zealand, and USA: A Systematic Review. J. Thorac. Dis..

[19] Kelly R. Too Deadly For Diabetes. https://toodeadlyfordiabetes.com.au/.

[20] Australian Indigenous HealthInfoNet Physical Activity—Programs. https://healthinfonet.ecu.edu.au/learn/determinants-of-health/health-behaviours/physical-activity/programs-and-projects/.

[21] Gidgup M.J.R., Kickett M., Hill K.D., Francis-Coad J., Weselman T., Coombes J., Ivers R., Bowser N., Palacios V., Hill A. (2022). Connecting and Reconnecting to a Community, with a Sense of Belonging—Exploring Aboriginal Elders’ Perspectives of Engaging in a Physical Activity Program. Health Promot. J. Aust..

[22] Lukaszyk C., Coombes J., Sherrington C., Tiedemann A., Keay L., Mackean T., Clemson L., Cumming R., Broe T., Ivers R. (2018). The Ironbark Program: Implementation and Impact of a Community-based Fall Prevention Pilot Program for Older Aboriginal and Torres Strait Islander People. Health Promot. J. Aust..

[23] Ivers R., Coombes J., Sherrington C., Mackean T., Tiedemann A., Hill A.-M., Keay L., Clemson L., Simpson J., Ryder C. (2020). Healthy Ageing among Older Aboriginal People: The Ironbark Study Protocol for a Cluster Randomised Controlled Trial. Inj. Prev..

[24] Kirwan M., Chiu C.L., Hay M., Laing T. (2021). Community-Based Exercise and Lifestyle Program Improves Health Outcomes in Older Adults with Type 2 Diabetes. Int. J. Environ. Res. Public Health.

[25] Kirwan M., Gwynne K., Laing T., Hay M., Chowdhury N., Chiu C.L. (2022). Can Health Improvements from a Community-Based Exercise and Lifestyle Program for Older Adults with Type 2 Diabetes Be Maintained? A Follow up Study. Diabetology.

[26] Kirwan M., Chiu C.L., Laing T., Chowdhury N., Gwynne K. (2022). A Web-Delivered, Clinician-Led Group Exercise Intervention for Older Adults With Type 2 Diabetes: Single-Arm Pre-Post Intervention. J. Med. Internet Res..

[27] Kirwan M., Chiu C.L., Fermanis J., Allison K., Laing T., Gwynne K. (2025). Culturally Adapted, Clinician-Led, Bilingual Group Exercise Program for Older Migrant Adults: Single-Arm Pre–Post-Intervention. Int. J. Environ. Res. Public Health.

[28] Kirwan M., Chiu C.L., Henson C., Laing T., Fermanis J., Scott L., Janszen J., Gwynne K. (2024). Empowering Through Group Exercise: Beat It Trainers’ Views on Successful Implementation of a Diabetes Management Program Online and In-Person. Diabetology.

[29] Lowitja Institute (2025). Co-Design Versus Faux-Design of Aboriginal and Torres Strait Islander Health Policy: A Critical Review.

[30] Kirkham R., Puszka S., Titmuss A., Freeman N., Weaver E., Morris J., Mack S., O’Donnell V., Boffa J., Dowler J. (2024). Codesigning Enhanced Models of Care for Northern Australian Aboriginal and Torres Strait Islander Youth with Type 2 Diabetes: Study Protocol. BMJ Open.

[31] Cameron D., Wilson A., Mendham A.E., Wingard S., Kropinyeri R., Scriven T., Kerrigan C., Spaeth B., Stranks S., Kaambwa B. (2024). Knowledge Interface Co-Design of a Diabetes and Metabolic Syndrome Initiative with and for Aboriginal People Living on Ngarrindjeri Country. Public Health Pract..

[32] Ni Mhurchu C., Te Morenga L., Tupai-Firestone R., Grey J., Jiang Y., Jull A., Whittaker R., Dobson R., Dalhousie S., Funaki T. (2019). A Co-Designed mHealth Programme to Support Healthy Lifestyles in Māori and Pasifika Peoples in New Zealand (OL@-OR@): A Cluster-Randomised Controlled Trial. Lancet Digit. Health.

[33] Kirwan M., Henson C., Bancroft-Duroux B., Meharg D., Christie V., Capes-Davis A., Boney S., Tully B., McCowen D., Ward K. (2026). Preventing Indigenous Cardiovascular Disease and Diabetes through Exercise (PrIDE) Study Protocol: A Co-Designed Wearable-Based Exercise Intervention with Indigenous Peoples in Australia. Diabetology.

[34] Gwynne K., Rambaldini B., Christie V., Meharg D., Gwynn J., Dimitropoulos Y., Parter C., Skinner J. (2022). Applying Collective Impact in Aboriginal Health Services and Research: Three Case Studies Tell an Important Story. Public Health Res. Pract..

[35] Kania J., Kramer M., Juster J., Ide S., List R.A., Anheier H.K., Toepler S. (2022). Collective Impact. International Encyclopedia of Civil Society.

[36] Bessarab D., Ng’andu B. (2010). Yarning About Yarning as a Legitimate Method in Indigenous Research. Int. J. Crit. Indig. Stud..

[37] National Health and Medical Research Council Ethical Guidelines for Research with Aboriginal and Torres Strait Islander Peoples. https://www.nhmrc.gov.au/research-policy/ethics/ethical-guidelines-research-aboriginal-and-torres-strait-islander-peoples.

[38] Lin I., Green C., Bessarab D. (2016). ‘Yarn with Me’: Applying Clinical Yarning to Improve Clinician–Patient Communication in Aboriginal Health Care. Aust. J. Prim. Health.

[39] Kirk M.A., Moore J.E., Wiltsey Stirman S., Birken S.A. (2020). Towards a Comprehensive Model for Understanding Adaptations’ Impact: The Model for Adaptation Design and Impact (MADI). Implement. Sci..

[40] Australian Government, Department of Health, Disability and Ageing Collection of Physical Activity and Sedentary Behaviour Guidelines for All Ages. https://www.health.gov.au/resources/collections/collection-of-physical-activity-and-sedentary-behaviour-guidelines-for-all-ages.

[41] Liguori G., Liguori G., Feito Y., Fountaine C.J., Roy B., American College of Sports Medicine, American College of Sports Medicine (2022). ACSM’s Guidelines for Exercise Testing and Prescription.

[42] Colberg S.R., Sigal R.J., Yardley J.E., Riddell M.C., Dunstan D.W., Dempsey P.C., Horton E.S., Castorino K., Tate D.F. (2016). Physical Activity/Exercise and Diabetes: A Position Statement of the American Diabetes Association. Diabetes Care.

[43] Chen G., Gully S.M., Eden D. (2001). Validation of a New General Self-Efficacy Scale. Organ. Res. Methods.

[44] The Heart Foundation Together, We Can Make Heart Disease History. https://www.heartfoundation.org.au/.

[45] The Heart Foundation Looking After Your Heart. https://www.heartfoundation.org.au/your-heart/aboriginal-health-risk-of-heart-disease.

[46] Harfield S., Pearson O., Morey K., Kite E., Canuto K., Glover K., Gomersall J.S., Carter D., Davy C., Aromataris E. (2020). Assessing the Quality of Health Research from an Indigenous Perspective: The Aboriginal and Torres Strait Islander Quality Appraisal Tool. BMC Med. Res. Methodol..

[47] Michie S., Atkins L., West R. (2014). The Behaviour Change Wheel: A Guide to Designing Interventions.

[48] Michie S., Richardson M., Johnston M., Abraham C., Francis J., Hardeman W., Eccles M.P., Cane J., Wood C.E. (2013). The Behavior Change Technique Taxonomy (v1) of 93 Hierarchically Clustered Techniques: Building an International Consensus for the Reporting of Behavior Change Interventions. Ann. Behav. Med..

[49] Michie S., Van Stralen M.M., West R. (2011). The Behaviour Change Wheel: A New Method for Characterising and Designing Behaviour Change Interventions. Implement. Sci..

[50] Teixeira P.J., Carraça E.V., Markland D., Silva M.N., Ryan R.M. (2012). Exercise, Physical Activity, and Self-Determination Theory: A Systematic Review. Int. J. Behav. Nutr. Phys. Act..

[51] Ryan R.M., Deci E.L. (2000). Self-Determination Theory and the Facilitation of Intrinsic Motivation, Social Development, and Well-Being. Am. Psychol..

[52] Department of the Prime Minister and Cabinet (2017). National Strategic Framework for Aboriginal and Torres Strait Islander Peoples’ Mental Health and Social and Emotional Wellbeing 2017–2023.

[53] Locke E.A., Latham G.P. (2002). Building a Practically Useful Theory of Goal Setting and Task Motivation: A 35-Year Odyssey. Am. Psychol..

[54] Koestner R., Lekes N., Powers T.A., Chicoine E. (2002). Attaining Personal Goals: Self-Concordance plus Implementation Intentions Equals Success. J. Pers. Soc. Psychol..

[55] Gollwitzer P.M., Sheeran P. (2006). Implementation Intentions and Goal Achievement: A Meta-analysis of Effects and Processes. Advances in Experimental Social Psychology.

[56] Fogarty W. (2018). Deficit Discourse and Strengths-Based Approaches: Changing the Narrative of Aboriginal and Torres Strait Islander Health and Wellbeing.

[57] Gwynne K., Jeffries T., Lincoln M. (2019). Improving the Efficacy of Healthcare Services for Aboriginal Australians. Aust. Health Rev..

[58] Ng J.Y.Y., Ntoumanis N., Thøgersen-Ntoumani C., Deci E.L., Ryan R.M., Duda J.L., Williams G.C. (2012). Self-Determination Theory Applied to Health Contexts: A Meta-Analysis. Perspect. Psychol. Sci..

[59] Gollwitzer P.M. (1999). Implementation Intentions: Strong Effects of Simple Plans. Am. Psychol..

[60] Aspinwall L.G., Taylor S.E. (1997). A Stitch in Time: Self-Regulation and Proactive Coping. Psychol. Bull..

[61] Tsey K., Whiteside M., Haswell-Elkins M., Bainbridge R., Cadet-James Y., Wilson A. (2010). Empowerment and Indigenous Australian Health: A Synthesis of Findings from Family Wellbeing Formative Research. Health Soc. Care Community.

[62] Sherwood J., Edwards T. (2006). Decolonisation: A Critical Step for Improving Aboriginal Health. Contemp. Nurse.

[63] Charles C., Gafni A., Whelan T. (1997). Shared Decision-Making in the Medical Encounter: What Does It Mean? (Or It Takes at Least Two to Tango). Soc. Sci. Med..

[64] CAST Universal Design for Learning Guidelines Version 3.0. https://udlguidelines.cast.org.

[65] Nash S., Arora A. (2021). Interventions to Improve Health Literacy among Aboriginal and Torres Strait Islander Peoples: A Systematic Review. BMC Public Health.

[66] Le Grande M., Ski C.F., Thompson D.R., Scuffham P., Kularatna S., Jackson A.C., Brown A. (2017). Social and Emotional Wellbeing Assessment Instruments for Use with Indigenous Australians: A Critical Review. Soc. Sci. Med..

[67] Bandura A. (2000). Exercise of Human Agency Through Collective Efficacy. Curr. Dir. Psychol. Sci..

[68] McLeroy K.R., Bibeau D., Steckler A., Glanz K. (1988). An Ecological Perspective on Health Promotion Programs. Health Educ. Q..

