# Supplementary material for: Operationalizing Co-Design in Exercise Interventions with Indigenous Peoples in Australia: Development and Cultural Adaptation of the PrIDE Tools
Source: Int J Environ Res Public Health. 2026 Feb 17;23(2):252. doi: 10.3390/ijerph23020252 (PMC12941342; doi:10.3390/ijerph23020252)
Supplement: Supplementary file 1 [file ijerph-23-00252-s001.zip › ijerph-4086310-supplementary.pdf]

Table S1. COM-B Analysis: How PrIDE Tools Address Behavior Change Mechanisms

COM-B Component	PrIDE Exercise Program	Strong Spirit Strong Self	Keep Your Heart Strong Educational Materials	Success Plan
**Psychological Capability** (knowledge and psychological skills)	Enhanced AEP cultural safety professional development; wearable technology for health literacy and self-monitoring; education on diabetes self-management, exercise, nutrition	Reconceptualizes capability to include collective efficacy, cultural knowledge, and spiritual strength; yarning with RA provides opportunity for social connection	Simplifies complex heart health information into three accessible messages; replaces clinical terminology with everyday language; designed to build health literacy through visual (brochure) and narrative (animation with voice-over) formats; aims to strengthen understanding of actionable health behaviors	Supports goal articulation (Q2), reflective planning (Q4), and decision-making skills (Q5); tool completion provides practice in planning capabilities
**Physical Capability** (physical skills and stamina)	Personalized and scaffolded exercise program with individualized progression; variety across aerobic, resistance, balance, flexibility; functional fitness assessment; provision of equipment and home resources	Assessment requires physical engagement; tool completion demonstrates capacity to participate	Not directly addressed by educational materials	Program selection (Q5) considers physical capabilities; barrier planning (Q4) accounts for physical constraints
**Social Opportunity** (interpersonal influences, cultural norms)	Recruitment through trusted ACCHOs/AMS; yarning-based delivery; group exercise option; fortnightly cook-ups; Elder involvement; cultural supervision; Indigenous RA	Explicitly values help-seeking from family, Elders, community; administration through yarning creates opportunity for social interaction; interdependence framed as strength	"Yarn with your clinic mob" framing positions health conversations with trusted providers as social connection; materials designed for sharing in group settings and social media; Facebook distribution enables peer discussion and social reinforcement;	Social strategies embedded in Q4: "ask family for help," "reach out to friend"; help-seeking framed as legitimate strategy

			culturally resonant artwork designed to create sense of community ownership	
**Physical Opportunity** (environmental context, resources)	Flexible delivery (group/individual choice); pre-program calls addressing transport, childcare, scheduling; simplified medical clearance; provision of equipment; wearable technology; home exercise resources	In-person administration removes separate appointment barrier; yarning accommodates diverse literacy levels; RA provides physical resources	Multiple distribution channels (printed brochure, animation, Facebook) accommodate diverse access needs and preferences; materials available without requiring clinic visits; brochure format portable for sharing with family	Q3 assesses physical opportunity preferences; Q4 systematically identifies and plans for physical barriers (transport, time, competing demands)
**Reflective Motivation** (conscious plans and evaluations)	Success Plan goal setting: co-designed resources designed to build ownership; strengths-based language; education via yarning; participant choice in format; 3-month timeframe designed to encourage sustainable thinking	Tool emphasizes belief in capability, effort-outcome expectations, problem-solving; wholistic framing connects to broader life goals; cultural framing positions capability as collective resource	Strengths-based framing ("Strong in spirit, Strong in body, and Strong in heart") emphasizes existing capability; three clear messages designed to support goal clarity and planning; positions small changes as achievable wins rather than overwhelming clinical targets; actionable guidance ("Eat well like our old people did") aims to support concrete planning	Q1 identifies intrinsic motivations; Q2 facilitates articulation of meaningful goals; Q3 honors autonomy; Q4 supports proactive coping; Q5 supports informed decision-making; aligns with Self-Determination Theory
**Automatic Motivation** (emotional responses, impulses, habits)	Indigenous artwork designed to create cultural resonance and belonging; real-time biofeedback provides immediate reinforcement; social reinforcement in groups; positive	Yarning creates emotionally safe experience; cultural framing designed to trigger positive associations; strengths-based language designed to evoke positive affect; spirit/culture/community	Indigenous artwork designed to create cultural resonance, belonging, and pride; vibrant culturally appropriate color palette designed to evoke positive	Strengths-based framing (Q2: "What would you feel proud of achieving?") designed to evoke positive emotions; options reflecting lived experiences

	affect from cook-ups; identity-based motivation from Indigenous-governed co-designed program	recognition designed to trigger identity-based motivation	affect; voice-over by Indigenous AEP provides relatable, trusted messenger; simplicity aims to reduce cognitive burden and increase appeal; visual storytelling designed to support emotional connection to health messages	designed to create sense of being understood; choice provision designed to provide autonomy satisfaction

Note: AEP = Accredited Exercise Physiologist; ACCHO = Aboriginal Community Controlled Health Organization; AMS = Aboriginal Medical Service; COM-B = Capability, Opportunity, Motivation— Behavior model; Q = Question; RA = Research Assistant. Note: This table reflects an analytical mapping of tool features to COM-B components based on theoretical alignment and design intent. It does not represent claims of demonstrated effectiveness, which will be evaluated during PrIDE implementation.
